# Niche differentiation among comammox (*Nitrospira inopinata*) and other metabolically distinct nitrifiers

**DOI:** 10.3389/fmicb.2022.956860

**Published:** 2022-09-14

**Authors:** Xueqin Yang, Xiaoli Yu, Qiang He, Ting Deng, Xiaotong Guan, Yingli Lian, Kui Xu, Longfei Shu, Cheng Wang, Qingyun Yan, Yuchun Yang, Bo Wu, Zhili He

**Affiliations:** ^1^Environmental Microbiomics Research Center, School of Environmental Science and Engineering, Southern Marine Science and Engineering Guangdong Laboratory (Zhuhai), Sun Yat-sen University, Guangzhou, China; ^2^Department of Civil and Environmental Engineering, The University of Tennessee, Knoxville, TN, United States; ^3^State Key Laboratory of Biocontrol, School of Ecology, Sun Yat-sen University, Guangzhou, China; ^4^College of Agronomy, Hunan Agricultural University, Changsha, China

**Keywords:** synthetic nitrifying community, niche differentiation, interaction, ammonia affinity, specific growth rate, substrate tolerance

## Abstract

Due to global change, increasing nutrient input to ecosystems dramatically affects the nitrogen cycle, especially the nitrification process. Nitrifiers including ammonia-oxidizing archaea (AOAs), ammonia-oxidizing bacteria (AOBs), nitrite-oxidizing bacteria (NOBs), and recently discovered complete ammonia oxidizers (comammoxs) perform nitrification individually or in a community. However, much remains to be learned about their niche differentiation, coexistence, and interactions among those metabolically distinct nitrifiers. Here, we used synthetic microbial ecology approaches to construct synthetic nitrifying communities (SNCs) with different combinations of *Nitrospira inopinata* as comammox, *Nitrososphaera gargensis* as AOA, *Nitrosomonas communis* as AOB, and *Nitrospira moscoviensis* as NOB. Our results showed that niche differentiation and potential interactions among those metabolically distinct nitrifiers were determined by their kinetic characteristics. The dominant species shifted from *N. inopinata* to *N. communis* in the N4 community (with all four types of nitrifiers) as ammonium concentrations increased, which could be well explained by the kinetic difference in ammonia affinity, specific growth rate, and substrate tolerance of nitrifiers in the SNCs. In addition, a conceptual model was developed to infer niche differentiation and possible interactions among the four types of nitrifiers. This study advances our understanding of niche differentiation and provides new strategies to further study their interactions among the four types of nitrifiers.

## Introduction

Nitrification is an important process in the nitrogen cycle, and it was widely believed as a two-step process for more than one century with ammonia oxidized to nitrite by ammonia-oxidizing bacteria (AOBs) or ammonia-oxidizing archaea (AOAs) (Könneke et al., 2005), and subsequently nitrite oxidization to nitrate by nitrite-oxidizing bacteria (NOBs). Complete ammonia oxidizers (comammoxs) were initially proposed according to optimal pathway length analysis (Costa et al., 2006), functionally enriched and finally isolated in recent efforts (Daims et al., 2015; van Kessel et al., 2015; Kits et al., 2017; Sakoula et al., 2021). The identification of comammox raises questions on their diversity and distribution in the environment. Metagenome and marker gene sequencing data analyses have demonstrated that newly discovered comammox populations are widely distributed in natural and engineered systems (Palomo et al., 2016; Pinto et al., 2016; Wang et al., 2017; Annavajhala et al., 2018; Orellana et al., 2018; Xia et al., 2018; Yang et al., 2020), which are highly overlapped with the habitat of AOAs, AOBs, and NOBs (Hatzenpichler, 2012; Prosser and Nicol, 2012; Stahl and de la Torre, 2012; Daims et al., 2016; Lawson and Lucker, 2018). These results indicate comammox is a common nitrifier in the environment, raising further questions about their niche differentiation and interactions in the environment (Daims et al., 2016; Santoro, 2016).

Niches of nitrifiers are highly overlapped as they could share similar habitats and substrates in the environment (Martens-Habbena et al., 2009). Cultivation studies of niche differentiation could reveal mechanisms about the origin and maintenance of species biodiversity, community assembly (HilleRisLambers et al., 2012), coexistence (File et al., 2012), species physiology (Hatzenpichler et al., 2008; Hatzenpichler, 2012), and habitat preferences (Bauer et al., 2018). For example, the high ammonia affinity of comammox indicated their prevalence at oligotrophic environments (Kits et al., 2017) and the cooperation possibility between anammox and AOAs determined by their ammonia affinity according to a biofilm model (Straka et al., 2019). As ammonia and nitrite are key substrates of nitrification (Falkowski et al., 2008; Canfield et al., 2010; Stein, 2015), of particular interest is niche differentiation among nitrifiers in response to ammonium concentrations, which may provide new insights into the biodiversity, ecophysiology, and evolution of metabolically distinct nitrifiers in the environment.

To identify patterns of niche differentiation and interactions of nitrifiers driven by ammonium, synthetic microbial ecology theories and approaches may provide a new strategy. Synthetic microbial communities are designed and built by microorganisms with known genome information, physiology, and metabolic characteristics in a well-defined medium (Johns et al., 2016). Natural environmental communities are generally complex and subjected to the influence of a multitude of environmental factors, making them difficult, if not impossible, to identify drivers of niche differentiation. While a major drawback of laboratory studies on pure cultures is the inherited inability to study population interactions and underlying mechanisms, which is critical to the manifestation of niche differentiation. To bridge such a gap, synthetic microbial ecology has recently been developed (Dolinsek et al., 2016; Lindemann et al., 2016; Zomorrodi and Segre, 2016; Lawson et al., 2019), and synthetic microbial communities have been used as a powerful tool to simplify natural microbial communities with reduced complexity and a controlled environment (Shou et al., 2007; Momeni et al., 2011; De Roy et al., 2014; Grosskopf and Soyer, 2014), which is promising to address niche differentiation of nitrifiers and their interactions in the environment.

In this study, we aimed to study niche differentiation among four types of nitrifiers and their underlying mechanisms in response to ammonium concentrations using synthetic nitrifying communities (SNCs). We used a bottom-up approach to construct SNCs with different combinations of *Nitrospira inopinata* (comammox), *Nitrososphaera gargensis* (AOA), *Nitrosomonas communis* (AOB), and *Nitrospira moscoviensis* (NOB) and examined their responses to comparable or beyond environmentally ammonium concentrations (0.2 to 20 mM). Our results showed that the niche differentiation of nitrifiers was largely driven by their ammonia affinity, specific growth rates, and ammonium/nitrite tolerance. This study advances our understanding of niche differentiation, coexistence, and interactions driven by ammonium among metabolically distinct nitrifiers in the environment.

## Materials and methods

### Selection of nitrifiers for synthetic nitrifying community construction

Synthetic microbial ecology theories and approaches (De Roy et al., 2014; Dolinsek et al., 2016; Johns et al., 2016) were used to select nitrifying strains. Genome information, function, representativeness, and physiology (e.g., growth medium, growth temperature, activity, and substrate concentration) of nitrifying strains (Prosser and Nicol, 2012) were considered for SNC construction. The isolate of comammox (*N. inopinata*, JCM 31988) was chosen as the representative of complete nitrifiers (Daims et al., 2015). Most AOAs were enriched and isolated from thermophilic and hyper-thermophilic environments (Hatzenpichler et al., 2008), while optimal temperatures for most AOBs were between 25 and 30°C (Itoh et al., 2013). To compromise the growth temperature for all nitrifiers, a moderately thermophilic AOA (*N. gargensis*, JCM 31473) was chosen (Hatzenpichler et al., 2008). Considering the growth temperature and substrate concentrations for culturing, we chose *N. communis* (DSMZ 28436) as AOB (Koops et al., 1991) as it is a less eutrophic AOB and could grow well at 37°C (Wu et al., 2013), and similarly, *N. moscoviensis* (provided by Eva Spieck from Hamburg University) was selected as NOB (Ehrich et al., 1995). The characteristics of those nitrifiers are summarized in Table 1.

**Table 1:** Characteristics of four nitrifiers.

**Characteristics**	***N. inopinata***	***N. gargensis***	***N. communis***	***N. moscoviensis***
Sources of strain	An oil exploration well	Hot spring	Soil	Heating water system
Cell morphology	Spiral-shaped cell with a flagellum	Round cell with a flagellum	Short rods or ellipsoidal with round ends	Irregularly shaped cells or spiral-shaped rods
Cell dimension (width × Length, μm)	0.18–0.3 × 0.7–1.6	Around 0.3 × 0.3	1.0–1.4 × 1.7–2.2	0.2–0.4 × 0.9–2.2
Optimal Temperature (°C)	37	46	28	39
Saturation constant for activity (K_m_)	0.65^a^; 372^b^	5.6^a^	Around 1,000^a^	9^b^
Maximum specific activity (V_max_)	12.8^c^;16.9^d^	28.7^c^	ND	18^d^
Saturation constant for growth (K_s_)	ND	ND	ND	ND
Maximum specific growth rate (μ_max_)	0.032 h^−1^	0.028 h^−1^	0.084 (0.055^e^	0.031 h^−1^

a μM (${\text{NH}}_{3}{\text{+NH}}_{4}^{+}$).

b μM ${\text{NO}}_{2}^{-}$.

c μM N per milligram protein per hour.

d μM ${\text{NO}}_{2}^{-}$ per milligram protein per hour.

e the growth of N. communis was denoted by OD_600_ after 3 days (1 day).

ND, not determined; characteristics were collected from previous articles (Ehrich et al., 1995; Spieck et al., 2006; Hatzenpichler et al., 2008; Spang et al., 2012; Daims et al., 2015; Nowka et al., 2015; Kits et al., 2017).

### Cultivation of nitrifiers

We took a step-by-step strategy to culture four nitrifiers individually, with a specific combination, or together. First, *N. inopinata* (37°C), *N. gargensis* (47°C), *N. communis* (28°C), and *N. moscoviensis* (37°C) were grown individually in their specific medium adding 1 mM, 1 mM, and 10 mM ammonium and 5.7 mM nitrite as their substrates, respectively, as previous studies described (Koops et al., 1991; Nowka et al., 2015; Palatinszky et al., 2015; Kits et al., 2017). Second, to determine the optimal temperature for culturing SNCs, nitrifier at a logarithmic phase was inoculated to its fresh medium over a wide range of temperatures (28 to 47°C for *N. inopinata* and *N. gargensis*, 16.5 to 40°C for *N. communis*, and 22 to 42.5°C for *N. moscoviensis*) (see Section Cultivation and construction of synthetic nitrifying communities). Since nitrifiers were sensitive to pH changes (He et al., 2012) and natural environment mainly is slightly alkaline, after several attempts at typical medium of nitrifiers, a mineral medium (Daims et al., 2015) with CaCO_3_ as buffer (pH = 7.8 after autoclaving) was selected for all SNCs culturing. Nitrifiers at the exponential phase were inoculated into a unified medium in 37°C with different substrates, and their performances were examined (see Section Cultivation and construction of synthetic nitrifying communities). Finally, nitrifiers at the exponential phase were inoculated to construct SNCs with an equal number of total cells (4 × 10^4^ cells/ml, see Section qPCR analysis of nitrifier dynamics of synthetic nitrifying communities). The medium with minor revision per liter, except the substrate (NH_4_Cl or NaNO_2_), contains 54 mg KH_2_PO_4_, 75 mg KCl, 50 mg MgSO_4_·7H_2_O, 584 mg NaCl, 4 g CaCO_3_, 1 ml of specific trace element solution (TES), and 1 ml of selenium–wolfram solution (SWS), as described previously (Daims et al., 2015). The pH was around 7.8 after autoclaving and during the growth as most undissolved CaCO_3_ in the medium was serving as the buffer solution. Nitrifiers were all grown in 100-ml bottles with 50 ml medium in the dark without shaking.

### Determination of optimal temperature and unified medium for synthetic nitrifying communities

To determine the optimal temperature for culturing, the dynamics of ammonium, nitrite, nitrate, and cell number were measured during the growth of *N. inopinata, N. gargensis, N. communis*, and *N. moscoviensis* at different temperature gradients under their optimal substrate concentrations, as described in Section Cultivation of nitrifiers (measurement methods in Section qPCR analysis of nitrifier dynamics of synthetic nitrifying communities and Section Determination of ammonium, nitrite, and nitrate concentrations). Specific ammonium/nitrite oxidation activity and growth rates were calculated for comparison (see Section Kinetics and statistical analysis). Similarly, performance of nitrifiers was determined at the unified medium under 37°C.

### Construction of synthetic nitrifying communities and analysis of their growth

We constructed one pure culture (i) N1 with *N. inopinata* only, and four SNCs (ii) N2A (*N. gargensis* and *N. moscoviensis*), (iii) N2B (*N. communis* and *N. moscoviensis*), (iv) N2C (*N. inopinata* and *N. moscoviensis*), and (v) N4 with all four types of nitrifiers. Cultivation was carried out at 37°C in a gradient of five ammonium concentrations (0.2, 1.0, 2.0, 10.0, and 20.0 mM). Samples from four biological replicates (*n* = 4) were taken every 12/24 h until ammonium was completely consumed or nitrification ceased (this longest experiment last 36 days, Supplementary Figure S1), followed by centrifugation (6,000 x g, 5 min, 4°C). The supernatant was stored at −20°C for ammonium, nitrite, and nitrate quantification; the pellets were washed with PBS two times, suspended with PBS solution, stored at −20 °C, and used for determining the dynamics of individual nitrifiers by real-time quantitative PCR (qPCR).

### qPCR analysis on nitrifier dynamics of synthetic nitrifying communities

The abundance of *N. inopinata, N. gargensis, N. communis*, and *N. moscoviensis* was determined by qPCR using newly designed gene-specific primer pairs (Supplementary Table S1). Reported genes like *amoA* and *nxrB* were not specific enough to distinguish them; therefore, we expand their whole genome to find single-copy specific gene for quantification. Strain-specific regions were obtained and filtered (Marcais and Kingsford, 2011; Tu et al., 2013), and then single copy genes were selected for primer design. Further specificity was checked by PCR, gel electrophoresis (Supplementary Figure S2), and qPCR analysis. Here, specificity means that a nitrifier in all SNCs could only be identified by its own primers. The relative abundance of nitrifiers was obtained by taking the maximum total cell number of SNCs under each ammonium concentrations at the endpoint as 100%. For example, the sum cell number of N4 under 0.2 mM at the endpoint was greater than that of other SNCs; thus, the relative abundance of each nitrifier at SNCs under 0.2 mM was converted by dividing its cell number by the maximum number, as well as for nitrifiers in SNCs under other concentrations.

Plasmids were obtained by PCR amplification of the target gene from DNA of pure cultures and cloning the product into a pEASY TA vector (TransGen, China), and were used as the standard for qPCR analysis, and the concentration of standards was determined by Qubit 4.0 (Thermo Fisher Scientific, USA). The amplification efficiency was between 90% and 110%, and the correlation coefficient (r^2^) of the standard curve was greater than 0.99.

The qPCRs were run with three technical replicates in a Bio-Rad C1000 CFX96 Real-Time PCR System (USA). Each qPCR was performed in a 12-μl reaction mix containing 6 μl SYBR Green Supermix (Bio-Rad, USA), 2.4 μl of the suspension, 0.3 μl of each primer (10 μM), and 3.3 μl of autoclaved double-distilled ultrapure water. Cells were lysed, and DNA was released for 3 min at 98°C, followed by 40 cycles of 15 s at 98°C, 30 s at 57.5°C, and 30 s at 72°C.

### Determination of ammonium, nitrite, and nitrate concentrations

Samples were collected during the experiment and centrifuged, and supernatants were used for ammonium, nitrite, and nitrate determination on a Skalar San^++^ Continuous Flow Analyzer (Netherlands) according to the manufacturer's instructions.

### Kinetics and statistical analysis

Specific ammonium/nitrite oxidation activity was determined by calculating the slope of log-transformed inverse ammonium (*N. inopinata, N. gargensis*, and *N. communis*) or nitrite (*N. moscoviensis*) concentrations against time (Kits et al., 2017). The specific ammonium/nitrite oxidation rate, growth rate, substrate affinity, free NH_3_ (FA) and free HNO_2_ (FNA), the Monod equation, and Haldane model were calculated (Supplementary Table S2) (Prosser and Nicol, 2012; Straka et al., 2019). Microbial community dissimilarity was analyzed by principal co-ordinates analysis (PCoA) with Bray–Curtis dissimilarity estimates, and the significance was tested by using the multiple-response permutation procedure (MRPP), analysis of similarities (ANOSIM), and permutational multivariate analysis of variance (ADONIS) (Martiny et al., 2011). The *t*-test, one-way analysis of variance (ANOVA), two-way ANOVA, and linear regression were performed by IBM SPSS 22 (SPSS Inc., USA).

## Results

### Cultivation and construction of synthetic nitrifying communities

**Figure 1:**
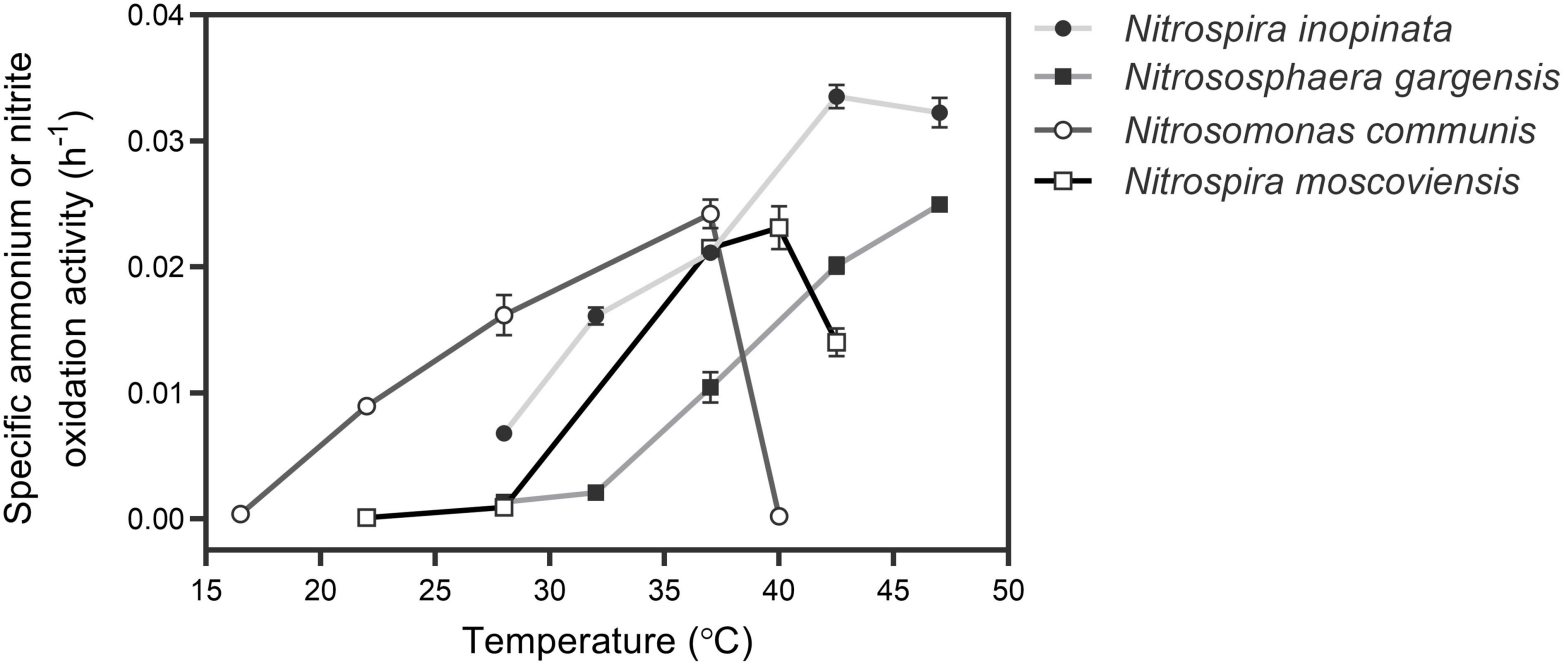
Determination of the optimal temperature for culturing four types of nitrifiers. For comammox (*N. inopinata*) and AOA (*N. gargensis*), the temperatures were 28, 32, 37, 42.5, and 47°C, respectively; for AOB (*N. communis*), the temperature range was from 16.5 to 40°C; for NOB (*N. moscoviensis*), the temperature was controlled from 22 to 42.5°C. Data points show mean ± SE (standard error, *n* = 4). If not visible, error bars are smaller than symbols.

**Figure 2:**
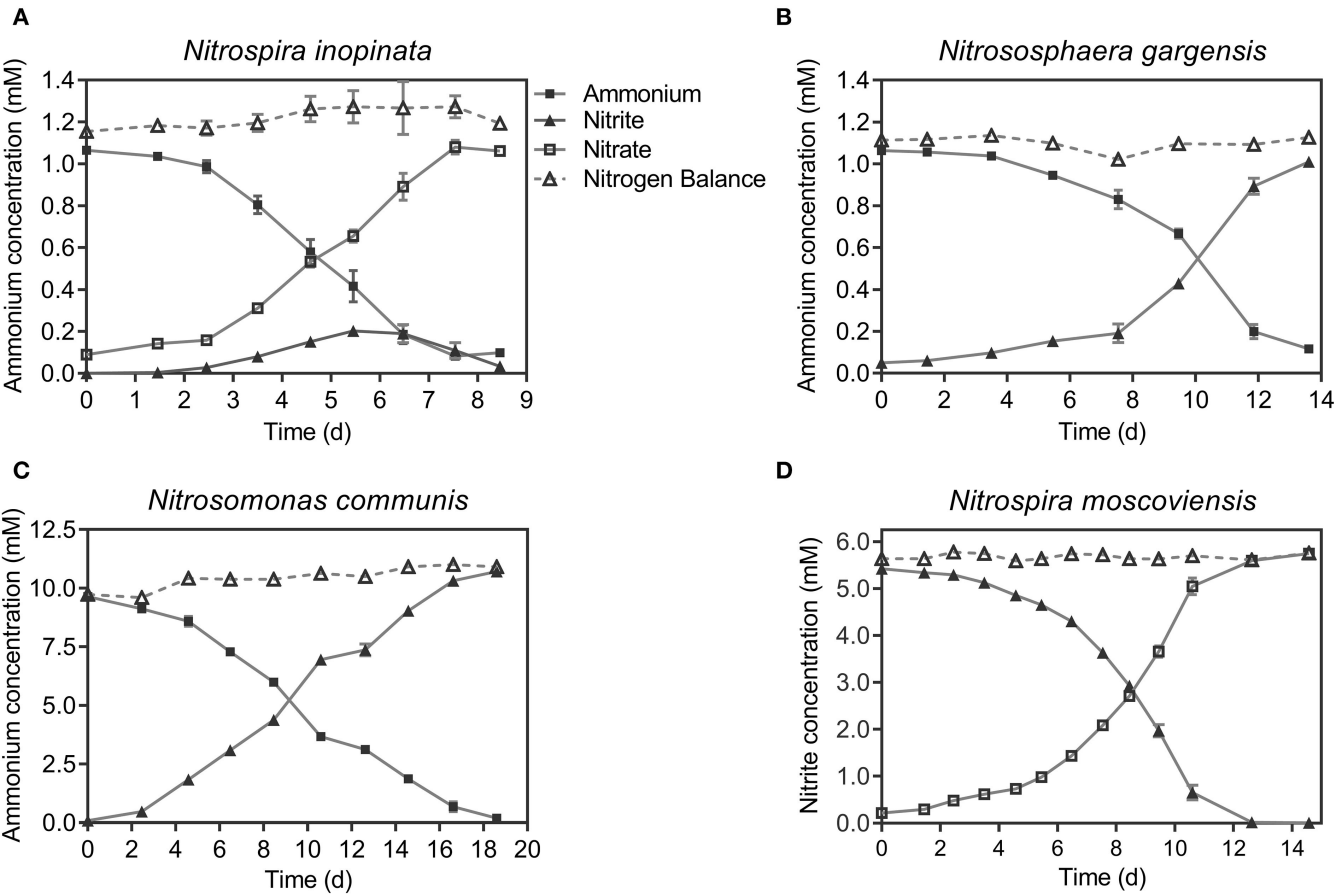
Ammonium or nitrite consumption and nitrite or nitrate production by nitrifiers grown at the unified medium at 37°C. **(A)** comammox (*N. inopinata*); **(B)** AOA (*N. gargensis*); **(C)** AOB (*N. communis*); **(D)** NOB (*N. moscoviensis*) at the exponential stage without quantifying the cell number were inoculated to fresh medium. The substrate concentrations were 1, 1, 10, and 5.7 mM, respectively. Data points are shown as mean ± SE (*n* = 5). If not visible, error bars were smaller than symbols.

To determine an appropriate temperature for culturing all SNCs, the specific ammonium or nitrite oxidation activity of four individual nitrifiers was measured with their specific media and optimal substrate (ammonium or nitrite) concentrations under a wide range of temperatures (Figure 1). Based on the sum of specific ammonium/nitrite oxidation activity of the four nitrifiers, we selected 37°C to culture those nitrifiers. Also, we unified the medium with a typical medium for ammonia oxidizers after several attempts by evaluating their performance (i.e., substrate consumption and nitrite/nitrate production) over time at 37°C. The results showed that four nitrifiers functioned in their corresponding nitrification processes (Figure 2), and their specific ammonium oxidation activity did not show differences with their optimal media at 37°C (Supplementary Table S3). To test niche differentiation of nitrifiers driven by ammonium based on the completeness of nitrification process, we constructed the N4 community (*N. inopinata, N. gargensis, N. communis*, and *N. moscoviensis*) and four other SNCs: N1 (*N. inopinata*), N2A (*N. gargensis* and *N. moscoviensis*), N2B (*N. communis* and *N. moscoviensis*), and N2C (*N. inopinata* and *N. moscoviensis*) for cross-validation, and those SNCs were cultivated at 37°C under the unified medium with a wide range of ammonium concentrations (0.2, 1, 2, 10, and 20 mM) in this study.

### Composition and structure shift of SNCs as a function of ammonium concentrations

The abundance of each nitrifier in the SNCs at the endpoint was quantified by qPCR to understand their niche differentiation driven by ammonium concentrations. Generally, principal co-ordinates analysis (PCoA) of those SNCs showed they significantly (*P* < 0.05) differed, except for N1 and N2C, when all ammonium concentrations were considered (Supplementary Figure S3; Supplementary Table S4), and N4 was significantly (*P* < 0.05) different from N1, N2A, N2B, and N2C under the gradient of ammonium concentrations, except N4 and N2B, when ammonium concentrations were 2 and 20 mM (Supplementary Figure S3; Supplementary Table S5).

**Figure 3:**
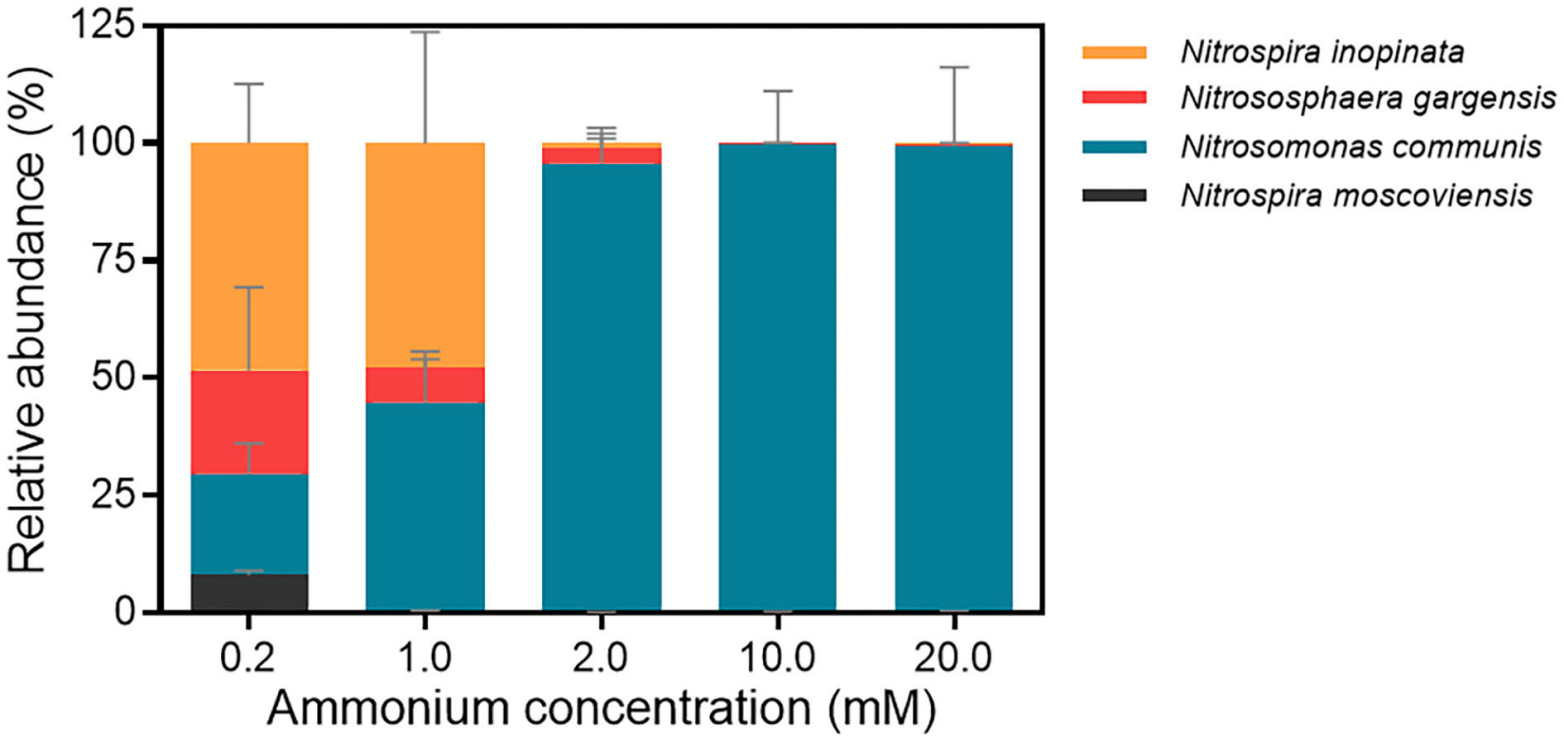
Relative abundances of four nitrifiers in the N4 community at end time point analyzed by qPCR. a. 0.2 mM; b. 1 mM; c. 2 mM; d. 10 mM; e. 20 mM. The end time point of each treatment was chosen when ammonium was completely consumed, or nitrification ceased. Data are presented as mean ± SE (*n* = 4).

We further analyzed the abundance dynamics of each nitrifier in the N4 community with the maximum cell (copy) number of four nitrifiers under each ammonium concentration as 100% (Figure 3; Supplementary Figure S4). The mean maximum total cell numbers under five ammonium concentrations were 1.22 × 10^5^, 3.77 × 10^6^, 1.89 × 10^7^, 1.53 × 10^8^, and 3.79 × 10^7^ copies/ml, respectively (Supplementary Table S6). All four nitrifiers coexist when ammonium concentration was 0.2 mM, and their cell number generally increased over time. At the final time point, *N*. *inopinata* was dominant (48.42%), followed by *N*. *gargensis* and *N*. *communis* with similar proportions (21.96% and 21.55%, respectively) and then *N. moscoviensis* (8.07%) (Figure 3). When the ammonium concentration increased to 1 mM, *N*. *inopinata* had a similar relative abundance with *N*. *communis* (47.70% and 44.44%, respectively), followed by *N*. *gargensis* (7.41%) and *N. moscoviensis* (0.44%) (Figure 3). When the ammonium concentration was 2 mM or higher, *N*. *communis* dominated the N4 community (95.61%−99.63%) (Figure 3). Therefore, the results indicated that *N*. *inopinata* was more competitive in the N4 community when ammonium concentrations were between 0.2 and 1 mM, while *N*. *communis* overwhelmed other nitrifiers when ammonium concentrations exceeded 2 mM, suggesting the importance of ammonium concentrations in the niche differentiation between *N*. *inopinata* and *N*. *communis*.

### Determinants of niche differentiation of nitrifiers

**Figure 4:**
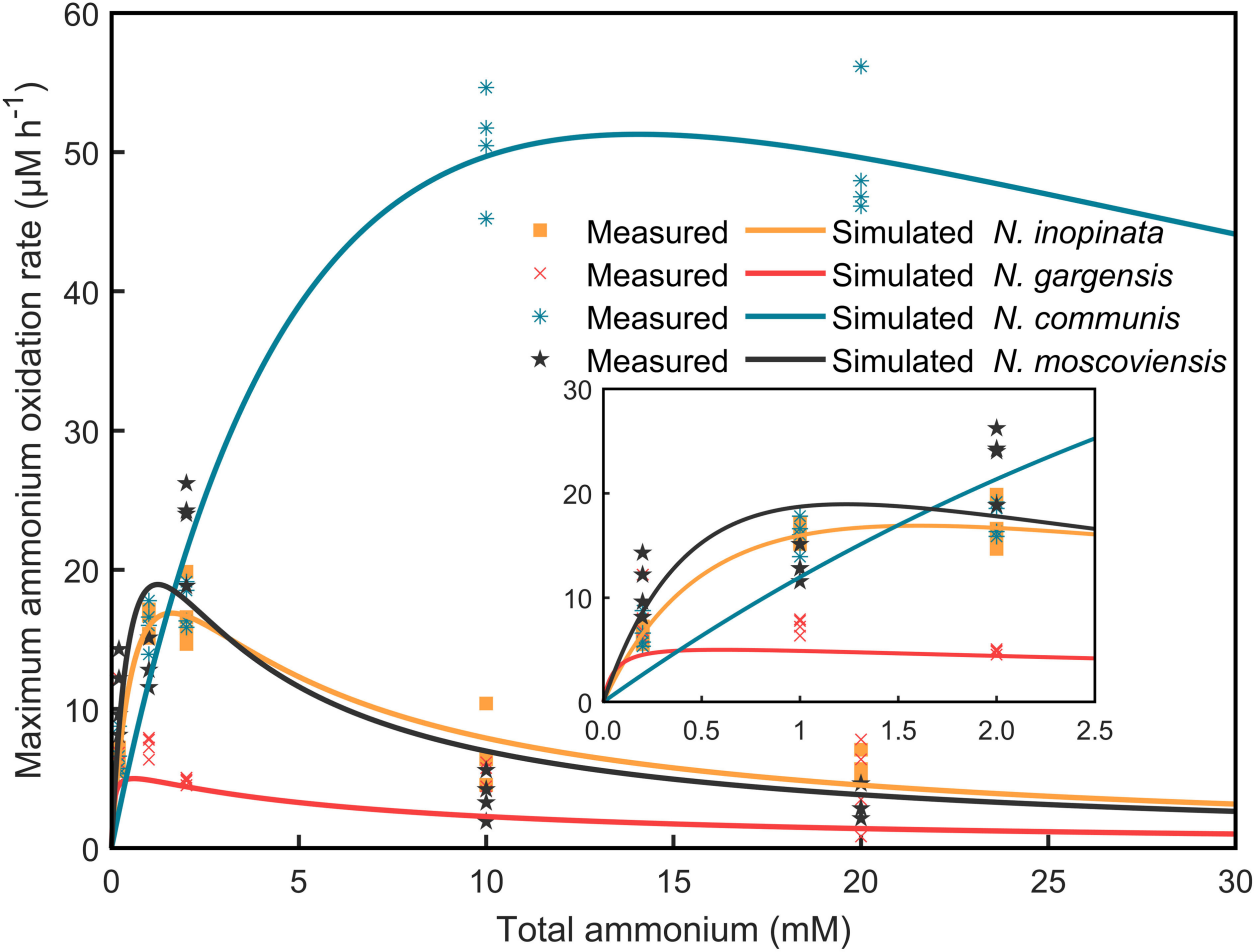
Specific ammonium/nitrite oxidation rates of four nitrifiers were fitted by using the Haldane model. The consumption of ammonium/nitrite against time in N1, N2A, N2B, and N2C communities at 0.2, 1, 2, 10, and 20 mM ammonium concentrations was extracted, and specific ammonium/nitrite oxidation rates were calculated and further fitted by using the Haldane model. The scatters mean the measured values with four replicates, while the solid lines show the fitted curve.

Given the population dynamics in response to ammonium concentrations in N4, it could be hypothesized that niche differentiation of nitrifiers would be driven by ammonia affinity, specific growth rate, and substrate tolerance as a function of ammonium concentrations, which could differentially impact each of those nitrifiers or SNCs. To test this hypothesis, we measured the dynamics of ammonium/nitrite/nitrate concentrations and abundances of nitrifiers. Furthermore, specific growth rates and ammonium/nitrite oxidation rates were analyzed and fitted by using the Haldane model (Supplementary Table S3; Figure 4).

**Figure 5:**
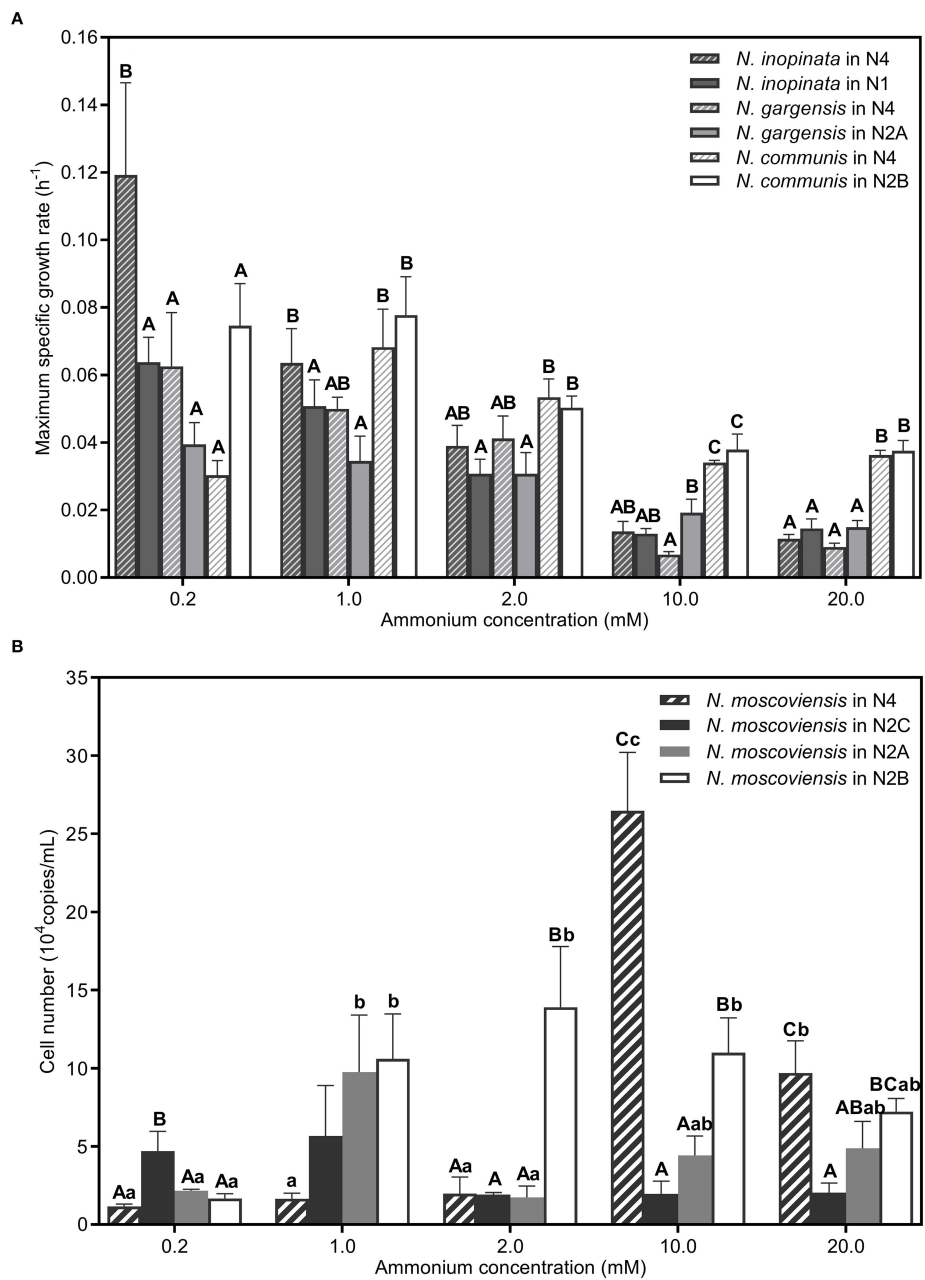
Maximum specific growth rates or cell numbers of nitrifiers in different SNCs. **(A)** Comparation of the maximum specific growth rate of *N. inopinata, N. gargensis*, and *N. communis* in different SNCs (N2A, N2B, N2C, and N4). **(B)** Cell numbers of *N. moscoviensis* in different SNCs (N2A, N2B, N2C, and N4). Data are presented as mean ± SE (*n* = 4). Different capital letters mean a statistical significance (*P* < 0.05) among the five ammonium concentrations for the same community, and different small letters mean a statistical significance (*P* < 0.05) among different communities under the same ammonium concentration.

***(i)Comammox (N. inopinata) predominance at low ammonium concentrations***. In the N4 community, the maximum specific growth rate of *N. inopinata* (0.119 h^−1^) was much higher than that of *N*. *gargensis* (0.063 h^−1^) and *N*. *communis* (0.030 h^−1^) when ammonium concentration was 0.2 mM (Figure 5A), which was consistent with their relative abundances (48.42%, 21.96%, and 21.55%, respectively) (Figure 3). Specifically, the maximum specific growth rate of *N. inopinata* and *N. gargensis* was significantly (*P* < 0.05) higher in N4 than that in N1 (0.119 vs. 0.064 h^−1^) or in N2A (0.063 vs. 0.039 h^−1^), respectively; the maximum specific growth rate of *N. communis* in N4 was much lower than that in N2B (0.030 vs. 0.075 h^−1^). Such growth differences suggested possible complex interactions among those nitrifiers in N4. Also, we used the Haldane model to fit their maximum specific ammonium oxidation rates with ammonium concentrations and calculated the mean K_m(app)_ of nitrifiers (Figure 4; Table 2). The value of K_m(app)_ was inversely proportional to ammonia affinity, showing that *N. gargensis* had the lowest K_m(app)_ (42 μM ${\text{NH}}_{4}^{+}$, R^2^ = 0.778), followed by *N. inopinata* (282 μM ${\text{NH}}_{4}^{+}$, R^2^ = 0.976) and *N. communis* (2,553 μM ${\text{NH}}_{4}^{+}$, R^2^ = 0.971), indicating that *N. gargensis* had the highest ammonia affinity, followed by *N. inopinata* and then *N. communis*. These results indicated that the competitiveness of comammox in the N4 community at 0.2 mM could be largely due to its high maximum specific growth rate and high ammonia affinity.

**Table 2:** Kinetic parameters of nitrifiers.

	**r_m_**	**K_S2_**	**K_I_**	**V_m_**	**K_m(app)_**	**R^2^**
*N. inopinata*	34.93^a^	857^b^	3,002^b^	16.88^a^	282^b^	0.976
*N. gargensis*	5.97^a^	58^b^	6,191^b^	5.00^a^	42^b^	0.778
*N. communis*	111.84^a^	8,310^b^	23,826^b^	51.28^a^	2,553^b^	0.971
*N. moscoviensis*	42.32^a^	763^c^	2,004^c^	18.94^a^	228^c^	0.732

The experimental data were calculated and modeled by using the Haldane model in N1, N2A, N2B, and N2C communities to show the relationship between specific ammonium/nitrite oxidation rates and ammonium concentrations.

a μM ammonium or nitrite h^−1^.

b μM total ammonium.

c μM nitrite.

***(ii)AOB predominance at high ammonium concentrations***. In the N4 community, the dominant species was shifted from *N. inopinata* to *N. communis* as ammonium concentrations increased (Figure 3). The specific growth rate of *N. communis* was higher than that of other nitrifiers when ammonium concentrations were 2 mM or above. Specifically, the maximum specific growth rate of *N. communis* (0.053 h^−1^) was much higher than that of *N. inopinata* (0.039 h^−1^) or *N. gargensis* (0.041 h^−1^) at 2 mM ammonium. As ammonium concentrations increased to 10 and 20 mM, the maximum specific growth rate of *N. communis* (0.034–0.036 h^−1^) was significantly (*P* < 0.05) higher than that of *N. inopinata* (0.011–0.014 h^−1^) or *N. gargensis* (0.007–0.009 h^−1^) (Figure 5A). The dramatic decrease in the growth of *N. inopinata* and *N. gargensis* indicated the effect of substrate inhibition, which was consistent with their relatively lower specific maximum ammonium oxidation rates at 10 and 20 mM ammonium (Figure 4). These results were consistent with the predominance of AOB (*N. communis*) in N2B at ammonium concentrations of 2 mM or above, likely due to high maximum specific growth rates of AOBs and a lack of inhibition by ammonium in the range of ammonium concentrations tested.

***(iii)Weak competitiveness and ammonium inhibition of AOA***. In the N4 community, the specific growth rate of *N. gargensis* decreased with the increase in ammonium concentrations (Figure 5A), which was consistent with the specific growth rates and ammonium oxidation rates observed for AOAs in N2A (Figure 4). At ammonium concentrations of 0.2 mM to 1 mM, ammonium was completely converted to nitrate, and the relative abundance of *N. gargensis* was 34.51% and 92.99%, respectively, while its abundance dramatically decreased to <1% when ammonium was 2 mM (Supplementary Figure S5). However, the maximum specific growth rate of *N. gargensis* in N2A was relatively low (0.015–0.019 h^−1^) (Figure 5A), and the concentrations of FA and FNA increased to 16.51 mg NH_3_-N/L and 0.040 mg HNO_2_-N/L, respectively, when the ammonium concentration was 10 mM. Therefore, these results indicated that the relatively slow growth of *N. gargensis* in the N4 community could be due to its unfavorable competition with *N. inopinata* (0.2 to 2 mM) and *N. communis* (2 mM), and ammonium inhibition at high ammonium concentrations (≥10 mM).

***(iv)Competition for nitrite and ammonium/nitrite inhibition of NOB***. The copy number was used to visualize the population dynamics of this NOB as the relative abundance of *N. moscoviensis* in the N4 community was too low to be visible in Supplementary Figures S4B–E. Ammonium oxidation to nitrite and increased maximum specific nitrite oxidation rates were observed in N2A and N2B at 0.2–1 mM and 0.2–2 mM ammonium, respectively (Supplementary Figures S1, S6), indicating that *N. moscoviensis* functioned well from 0.2 to 2 mM ammonium. The results were also supported by the increase in *N. moscoviensis* from 0.2 mM to 1 mM (2.18 × 10^4^-9.76 × 10^4^ copies/ml) in N2A and from 0.2 mM to 2 mM (1.66 × 10^4^-1.39 × 10^5^ copies/ml) in N2B (Figure 5B). However, the cell number of *N. moscoviensis* was much lower (1.16–1.98 × 10^4^ copies/ml) in N2C, especially in N4, than that in N2A or N2B from 0.2 to 2 mM, indicating a possible competition for nitrite between *N. moscoviensis* and *N. inopinata*. Also, the cell number of *N. moscoviensis* decreased significantly (*P* < 0.05) from 10 to 20 mM in N4, and similar results were observed in N2A and N2B (Figure 5B), suggesting ammonium/nitrite inhibition of *N. moscoviensis* growth at high ammonium concentrations. These results indicated the niche differentiation of *N. moscoviensis* was compromised by *N. inopinata* at 0.2 to 2 mM ammonium and inhibited by high ammonium/nitrite concentrations.

## Discussion

Understanding the niche differentiation, interaction, and coexistence of nitrifiers and their associated mechanisms is a central issue in microbial ecology. Our current knowledge of nitrifiers is generally based on limited monocultures and environmental studies, and their coexistence, niche differentiation, and interactions in the environment are extremely difficult or impossible to address. As various differences in physiology of monocultures exist, it is well inferred that substrate (ammonium and nitrite), temperature, pH, and H_2_O_2_ detoxification are considered the main drivers of niche differentiation among metabolically distinct nitrifiers (Prosser and Nicol, 2012; Hu and He, 2017; Kits et al., 2017). The physiology and diversity of cultivated AOAs indicated that they had a wider range of temperature and pH adaption than most AOBs (Lehtovirta-Morley et al., 2011; Zhang et al., 2017; Prosser et al., 2020; Picone et al., 2021); α-keto acids (e.g., pyruvate) which were reported to enhance the growth of some AOAs (Tourna et al., 2011; Qin et al., 2014) were further confirmed as H_2_O_2_ scavengers (Kim et al., 2016). Recently, several studies further indicated that nitrifiers were metabolically versatile beyond the N cycle and involved in hydrogen and sulfide oxidation (Lehtovirta-Morley et al., 2011; Tourna et al., 2011; Stahl and de la Torre, 2012; Daims et al., 2016). Among such many drivers, we gave priority to answering niche differentiation to ammonium as it is the energy source for nitrifiers. Thus, in this study, we applied synthetic microbial ecology theories and approaches to construct SNCs under unified medium and temperature and tested niche differentiation with comammox and other three types of metabolically distinct nitrifiers under a gradient of environmentally comparable ammonium concentrations to reflect the performance of nitrifiers in the environment. We found that those nitrifiers could coexist at low ammonium concentrations, and their niches differentiated under different ammonium concentrations with possible mechanisms, including ammonia/nitrite affinity, specific growth rate, and ammonium/nitrite tolerance. In addition, the results allowed us to explore possible interactions among those nitrifiers. Specifically, *N. inopinata* was the most competitive population at low ammonium concentrations, and *N. communis* was dominant at high ammonium concentrations. While *N. gargensis* and *N. moscoviensis* were less competitive than *N. inopinata* and *N. communis* for ammonium and *N. inopinata* for nitrite, respectively, at our tested ammonium ranges, the growth of *N. inopinata, N. gargensis*, and *N. moscoviensis* were inhibited at high ammonium concentrations.

First, ammonia/nitrite affinity of nitrifiers is the most important theoretical basis of niche differentiation at low ammonium environments (Hatzenpichler, 2012; Prosser and Nicol, 2012; Kits et al., 2017). For ammonia affinity, how AOAs and AOBs establish their niches and function in the environment has been discussed for decades, and comammox has recently joined in this debate. Previous laboratory studies showed that *N. inopinata* had the highest ammonia affinity, followed by non-marine AOAs and AOBs (Kits et al., 2017), which was reflected in the apparent half-saturation constant value [K_m(app)_] of *N. inopinata, N. gargensis*, and *Nitrosomonas* AOBs at 0.65, 5.6, and ~1,000 μM total ammonium, respectively (Martens-Habbena et al., 2009; Kits et al., 2017). Comammox populations have been considered as oligotrophs and could be prevalent at low ammonium environments, which is evidenced by many studies with microbial enrichments or environmental samples from oligotrophic environments, such as drinking water systems, rapid gravity sand filter, and groundwater-fed rapid sand filter (Palomo et al., 2016; Kits et al., 2017; Pjevac et al., 2017; Fowler et al., 2018; Xia et al., 2018). Also, AOAs were reported to have an advantage over AOBs in oligotrophic environments like open ocean (Könneke et al., 2005; Prosser and Nicol, 2008; Santoro et al., 2008; Walker et al., 2010), while eutrophic environments (e.g., fertilized agricultural soils) favored AOBs (Jia and Conrad, 2009; Prosser and Nicol, 2012; Hink et al., 2018). In this study, we found that the dominant species in N4 was *N. inopinata*, followed by *N. gargensis* and *N. communis* at low ammonium concentrations. *N. gargensis* exhibited the lowest K_m(app)_, followed by *N. inopinata* and *N. communis* (282 and 2,553 μM total ammonium, respectively). Such relatively high numerical values of K_m(app)_ observed may be due to higher ammonium concentrations used in this study than in previous physiological studies, and the contrast of K_m(app)_ between *N. inopinata* and *N. gargensis* could also result from the low activity and growth of *N. gargensis* in our experiments, thus affecting the degree of model fitting. These results generally support the predominance of comammox at low ammonium concentrations due to its high ammonia affinity, which is consistent with previous studies that comammox preferred oligotrophic lifestyle (Palomo et al., 2016; Pinto et al., 2016; Kits et al., 2017; Fowler et al., 2018; Xia et al., 2018). For nitrite affinity, known NOBs are affiliated with seven genera (Daims et al., 2016), of which *Nitrospira* NOBs are considered as *K* strategists and *Nitrobacter* NOBs as *r-*strategists (Nowka et al., 2015), and coincidently, all known comammoxs belong to *Nitrospira*. Nevertheless, the K_m_(app) of *N. inopinata* to nitrite was 372 μM, close to that of *Nitrobacter* NOB (Kits et al., 2017), which was higher than *Nitrospira* NOB. In this study, we found that the K_m_(app) of *N. moscoviensis* was 228 μM nitrite, which was lower than that of *N. inopinata*, indicating a niche of *N. inopinata* and *N. moscoviensis* in nitrite oxidation; thus, *N. inopinata* may prefer higher nitrite concentrations than *N. moscoviensis*.

Second, the ammonium/nitrite inhibition of nitrifiers may play important roles in their niche differentiation (Prosser and Nicol, 2012) as the growth and activity of nitrifiers were inhibited by different concentrations of FA and FNA (Anthonisen et al., 1976; Liu et al., 2019). A previous study showed that FA inhibition concentrations of AOB and NOB were 10–150 mg NH_3_-N/L and 0.1–1.0 mg NH_3_-N/L, respectively (Anthonisen et al., 1976). Also, the oxidation rate of *Nitrosomonas* and *Nitrobacter* decreased when FNA concentrations reached 0.10 (Vadivelu et al., 2006) and 0.011 mg HNO_2_-N/L, respectively, and was completely inhibited at the FNA concentration of 0.40 mg and 0.023 mg HNO_2_-N/L, respectively (Vadivelu et al., 2007), which are consistent with the results of this study, showing that high ammonium concentrations inhibited the growth of *N. inopinata* and *N. moscoviensis*, but no obvious inhibition was observed for *N. communis*. In addition, we found 2 mM ammonium would inhibit both ammonium oxidation and growth of AOA, which agrees with a previous study that shows an inhibition concentration of 3.08 mM ammonium (Hatzenpichler et al., 2008). Therefore, our results indicated that the ammonium/nitrite tolerance was different among these nitrifiers, which could explain the dominance of AOBs at high ammonium concentrations.

Third, the specific growth rate of nitrifiers under different ammonium concentrations may provide new insights into our understanding of niche differentiation. Generally, the growth rate of nitrifiers against substrate concentrations is described by the Monod equation (Prosser and Nicol, 2012). Previous studies showed that AOBs had higher specific growth rates than AOAs (Prosser and Nicol, 2012; Terada et al., 2013), and comammox nitrifiers were predicted to have a lower specific growth rate than canonical ammonia oxidizers (Costa et al., 2006). Kits et al. (2017) reported that the maximum specific ammonia oxidation activity of *N. inopinata* and *N. gargensis* was 0.032 h^−1^ (37°C) and 0.028 h^−1^ (46°C), respectively, and the specific ammonia oxidation activity of *N. gargensis* was 0.014 h^−1^ at 37°C. In this study, we found that *N. communis* had the highest maximum specific growth rate, followed by *N. inopinata, N. moscoviensis*, and *N. gargensis* from N1, N2A, and N2B, respectively, which was different from the prediction of comammox (Costa et al., 2006). However, the maximum specific growth rates of nitrifiers were different in N4, with the highest maximum specific growth rate for *N. inopinata*, followed by *N. communis, N. gargensis*, and *N. moscoviensis*, indicating complex interactions among metabolically distinct nitrifiers. Such interactions and underlying mechanisms need to be further explored in future.

**Figure 6:**
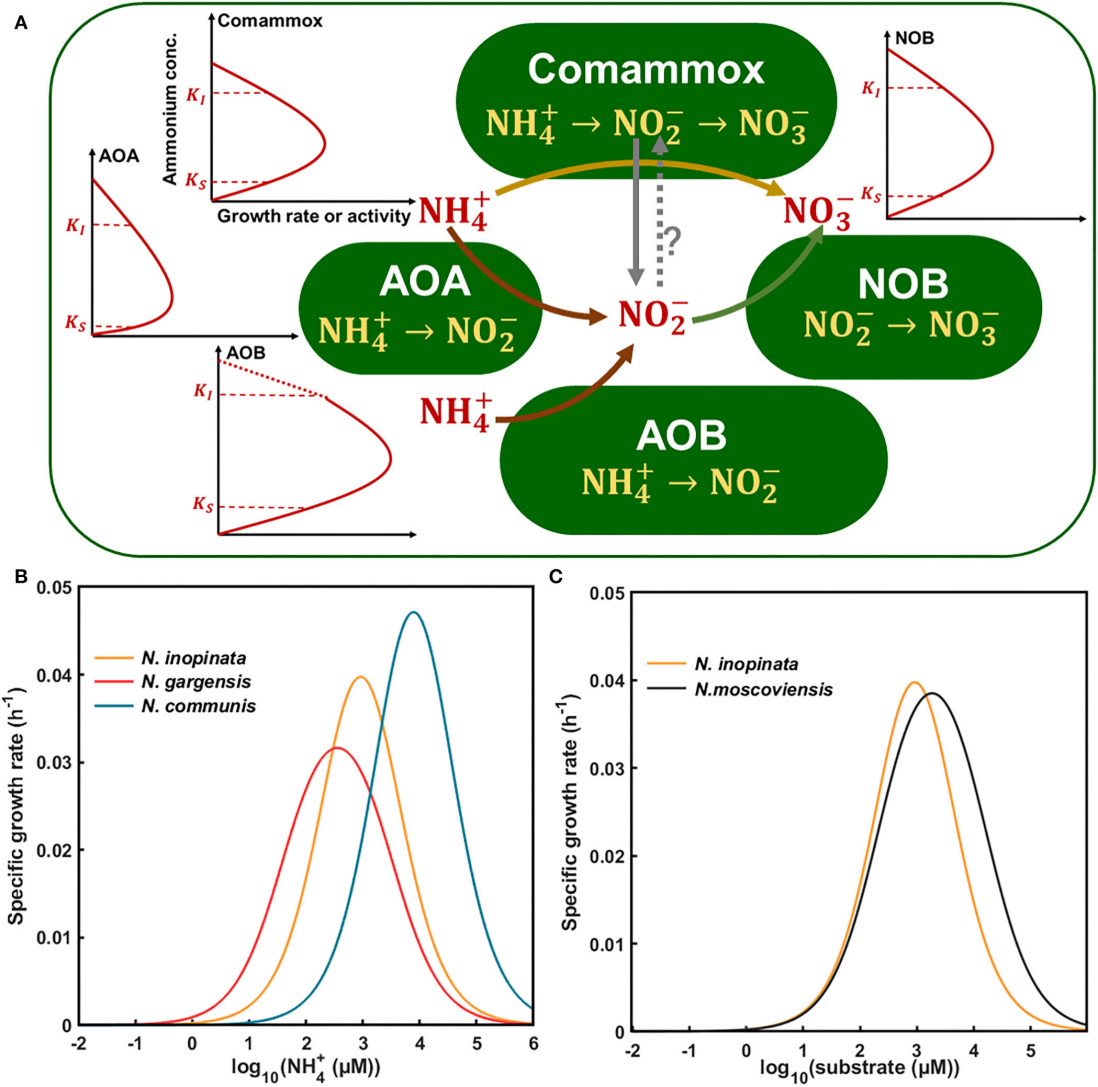
Conceptual framework for kinetics-driven niche differentiation of four nitrifiers (*N. inopinata, N. gargensis, N. communis*, and *N. moscoviensis*) in response to ammonium or nitrite. **(A)** Kinetics of nitrifiers including ammonia affinity, maximum specific growth rate or activity, and substrate inhibition under a wide gradient of ammonium concentrations. Words in red represent extracellular or environmental substances, and words in yellow represent intracellular metabolic pathways for nitrification processes with ammonium as substrate. The solid lines with arrows show the direction of pathways, and the dotted line with arrows and a question mark means uncertain. The red solid lines represent the growth or activity of nitrifiers in response to substrate concentrations, and the dotted part of line of AOB is a theoretical extension for potential inhibition at high ammonium concentrations as no obvious inhibition was observed in this study. **(B)** Niche differentiation of *N. inopinata, N. gargensis*, and *N. communis* in response to a wide gradient of ammonium concentrations based on our study. **(C)** Niche differentiation of *N. inopinata* and *N. moscoviensis* in response to nitrite with ammonium as substrate. K_S_ was represented by K_m(app)_ in Table 2 (282, 42, 2,553, and 228 μM total ammonium or nitrite, respectively); K_I_ of *N. inopinata* and *N. communis* was from Table 2, and K_I_ of *N. gargensis* and *N. moscoviensis* was obtained from the literature and modified (Hatzenpichler et al., 2008; Nowka et al., 2015). The specific growth rates of nitrifiers from N1, N2A, and N2B communities (0.064, 0.039, 0.078, and 0.048 h^−1^) were used for the Haldane model.

Based on the aforementioned results and current knowledge, we developed a conceptual model to understand kinetics-driven niche differentiation among those nitrifiers. It may be generally assumed that comammox had the highest ammonia affinity, followed by non-marine AOAs and AOBs, and NOBs had higher nitrite affinity than comammox when ammonium was used as the substrate. AOBs had the highest maximum specific growth rate or activity, followed by comammoxs, NOBs, and AOAs. The growth or activity of nitrifiers was inhibited by ammonium for AOAs, comammoxs, and AOBs sequentially, and NOBs appeared to tolerate higher nitrite than comammoxs. It is also noted that nitrite produced by *N. inopinata* (comammox) may be converted in the periplasm (Daims et al., 2015), and nitrite may leak out of comammox cells for NOB growth, as observed in this study, which may relieve its ammonium/nitrite inhibition and entail NOB abundance, which were higher in N4 at high ammonium concentrations (Figure 5B). Also, we used the aforementioned kinetics parameters (e.g., ammonia affinity, maximum specific growth rate, and ammonium oxidation rate) to construct the niche differentiation model of nitrifiers (Figure 6A), predicting that comammox might be dominant at low and moderate ammonium concentrations, and AOBs at high ammonium concentrations, which was different from our hypothesis. However, the chosen temperature was not optimal for AOAs, and the tested ammonium concentrations appeared not to be sufficiently low, preventing us from observing the predominance of AOAs at the lowest ammonium concentration tested in this study (Figure 6B). Our results showed that NOBs would utilize nitrite leaked by comammox when ammonium was high (e.g., 2 mM). The niche differentiation of comammoxs and NOBs for nitrite oxidation with ammonium as substrate was predicted (Figure 6C), and NOBs would also take leaked nitrite from comammox for growth, indicating their complex interactions. These results indicated that ammonia affinity, substrate inhibition, and specific growth rates could drive niche differentiation and potential interactions among metabolically distinct nitrifiers in response to ammonium concentrations in the environment.

## Conclusion

In summary, we constructed SNCs with four types of nitrifiers to understand niche differentiation in response to ammonium concentrations and found that niche differentiation was driven by ammonia affinity, specific growth rate, and ammonium/nitrite tolerance under a gradient of ammonium concentrations among those nitrifiers. In the N4 community, the dominant species was shifted from comammoxs to AOBs as ammonium concentrations increased. Comammox predominated at low ammonium concentrations due to its high ammonia affinity and high maximum specific growth rates. AOB predominated at high ammonium concentrations due to its high maximum specific growth rate, high ammonium oxidation rate and no obvious inhibition by ammonium. AOA had constantly low growth rates due to its weak competitiveness with comammox (0.2 to 1 mM) and AOB (2 mM), and inhibition by high ammonium concentrations (≥10 mM). Niche differentiation of NOBs could be compromised by comammox at 0.2 to 2 mM ammonium and inhibited by high ammonium/nitrite concentrations. Although the mechanism of nitrifier interactions and trade-offs between ammonium concentration and abundance detection sensitivity needs to be further explored, this study advances our understanding of niche differentiation of nitrifiers in response to ammonium concentrations in the environment, and it has important implications for exploring interactions among metabolically distinct nitrifiers using the established SNCs and multi-omics technologies.

## Data availability statement

The original data presented in the study are included in the article/Supplementary material, further inquiries can be directed to the corresponding authors.

## Author contributions

XYa, BW, and ZH conceptualized the research. XYa performed experiments and analyzed data with contributions from XYu, XG, and TD. XYa, QH, and ZH led the writing with contributions from all the other authors. All authors contributed to the article and approved the submitted version.

## Funding

This work was supported by the National Natural Science Foundation of China (31770539, 91951207) and by the Southern Marine Science and Engineering Guangdong Laboratory (Zhuhai) (SML2020SP004, 311021006).

## Conflict of interest

The authors declare that the research was conducted in the absence of any commercial or financial relationships that could be construed as a potential conflict of interest.

## Publisher's note

All claims expressed in this article are solely those of the authors and do not necessarily represent those of their affiliated organizations, or those of the publisher, the editors and the reviewers. Any product that may be evaluated in this article, or claim that may be made by its manufacturer, is not guaranteed or endorsed by the publisher.
