# Supplementary material for: Niche differentiation among comammox (Nitrospira inopinata) and other metabolically distinct nitrifiers
Source: Front Microbiol. 2022 Sep 14;13:956860. doi: 10.3389/fmicb.2022.956860 (PMC9515657; doi:10.3389/fmicb.2022.956860)
Supplement: Supplementary file 1 [file Data_Sheet_1.docx]

**Niche differentiation among comammox (*Nitrospira inopinata*) and other** **metabolically distinct nitrifiers**

**Supporting Information**

1. **Supplementary Tables**

**Table S1** Specific primer pairs designed for specifically identifying each nitrifier in the synthetic nitrifying communities

**Table S2** Kinetics calculation equations

**Table S3** Specific ammonium or nitrite oxidation activity at 37℃ under their optimal media and the unified medium

**Table S4** Analysis of similarities (ANOSIM) among N1, N2C, N2A, N2B and N4 communities at the final timepoint under different ammonium concentrations

**Table S5** Multiple-response permutation procedure (MRPP) analysis between N4 and other four communities at the final time point under different ammonium concentrations

**Table S6** The maximum total cell number in five ammonium concentrations

1. **Supplementary figures**

**Figure S1** The ammonium, nitrite and nitrate concentrations in N1, N2C, N2A, N2B and N4 communities under five (0.2 to 20 mM) ammonium concentrations

**Figure S2** The primer specificity for quantitative PCR (qPCR) analysis of four nitrifiers

**Figure S3** Principal co-ordinates analysis (PCoA) of synthetic nitrifying communities (N1, N2A, N2B, N2C and N4) at five ammonium concentrations at the last time point

**Figure S4** Relative abundances of four nitrifiers in the N4 community analyzed by qPCR**.** a. 0.2 mM; b. 1 mM; c. 2 mM; d. 10 mM; e. 20 mM

**Figure S5** The abundance (a-e) and ammonium oxidation (f-j) of N2A (*N. gargensis* and *N. moscoviensis*) under five (0.2 to 20 mM) ammonium concentrations

**Figure S6** Maximum ammonium and nitrite oxidation rates of five nitrifying communities at the ammonium concentrations of 0.2, 1, 2, 10 and 20 mM

**Table S1** Specific primer pairs designed for specifically identifying each nitrifier in the synthetic nitrifying communities.

| **Microorganism**  **(Accession number)** | **5’ - 3’** | **Genome site** | **length** |
| --- | --- | --- | --- |
| *N. inopinata*  (NZ_LN885086) | TCGAAATCTCCAACGTGGACAT | 2839679-2839770, part of an open reading frame | 92 bp |
|  | AGTTTTCATCGTTCGCAGGTTG |  |  |
| *N. gargensis*  (NC_018719) | TATCAAGGCCAAGCTCGAGC | 2075439-2075590, part of an open reading frames | 152 bp |
|  | GGCACCATCCTGAAGAACCA |  |  |
| *N. communis*  (NZ_CP011451) | TGCTCTCTGGCGGAGACTTA | 2042654-2042746, part of an open reading frame | 93 bp |
|  | TGCAGGTGCCACTACATGAG |  |  |
| *N. moscoviensis*  (NZ_CP011801) | TGTCGTGATCGCTCAGATGG | 1559399-1559510, out of two open reading frames | 112 bp |
|  | CCTGCACGTGTCACCACTAT |  |  |

***** The qPCR reactions were run with three technical replicates in a Bio-Rad C1000 CFX96 Real-Time PCR system (USA). Each qPCR reaction was performed in a 12-μl reaction mix containing 6 μl SYBR Green Supermix (Bio-Rad, USA), 2.4 μl of the suspension, 0.3 μl of each primer (10 μM), and 3.3 μl of autoclaved double-distilled ultrapure water. Cells were lysed, and DNA was released for 3 min at 98°C, followed by 40 cycles of 15 s at 98°C, 30 s at 57.5°C, and 30 s at 72°C

**Table S2** Kinetics calculation equations.

| **Kinetics** | **Equation** |
| --- | --- |
| Specific ammonium/nitrite oxidation rate (*v*) | $v = \frac{S_{2}-S_{1}}{t_{2}-t_{1}}$ (Eq.1),  where S (μM) is substrate concentration (ammonium or nitrite); t (h) is time; v (μM/h) is the specific substrate oxidation rate. |
| Specific growth rates (μ) | $\mu= \frac{\ln\left( N_{2} \right)-{ln(N}_{1})}{t_{2}-t_{1}}$ (Eq.2),  where N (copies/mL) is the cell number of nitrifier; μ (h^-1^) is the specific growth rate; t (h) is time as described in Eq. 1. |
| Specific substrate affinity (a^0^) | $a^{0}=\frac{V_{\max}}{K_{m}}$ (Eq.3),  where a^0^ (g wet cells^-1^ h^-1^) is the specific substrate affinity; v_max_ is the maximum rate (g substrate/g wet cells^-1^ h^-1^); K_m_ is the half saturation constant (g substrate) (Kits et al., 2017). |
| The free NH_3_ (FA) | $FA=\frac{17}{14}\times\frac{S(\mathrm{NH}_{4}^{+}-N)\times{10}^{\mathrm{pH}}}{e^{(\frac{6344}{273+T})}+{10}^{\mathrm{pH}}}$ (Eq.4),  where FA (mg NH_4_^+^-N/L) is the free NH_4_^+^ concentration; T (°C) is the temperature; S (μM) is described in Eq. 1 (Anthonisen et al., 1976) |
| The free HNO_2_ (FNA) | $FNA=\frac{46}{14}\times\frac{S(\mathrm{NO}_{2}^{-}-N)}{e^{(\frac{-2300}{273+T})}{\times10}^{\mathrm{pH}}}$ (Eq.5),  where FNA (mg NO_2_^-^-N/L) is NO_2_^-^ concentration; S (μM) and T (°C) are described in Eq. 4 (Anthonisen et al., 1976). |
| The Monod equation | $\mu= \frac{\mu_{\max}S}{K_{S1}+S}$ (Eq.6),  where K_S1_ (μM NH_3_+NH_4_^+^) is the half-saturation constant for growth; μ_max_ (h^-1^) is the maximum specific growth rate; S (μM) is described in Eq. 1 (Prosser and Nicol, 2012). |
| The Haldane Model | $r=\frac{r_{\max}S}{K_{S2}+S+\frac{S^{2}}{K_{I}}}$ (Eq.7),  where r_max_ (μM/h), K_S2_ (μM NH_3_+NH_4_^+^), and K_I_ (μM NH_4_^+^ or NO_2_^-^) are the maximum specific substrate conversion rate, saturation constant, and substrate inhibition concentration, respectively fitted by the Haldane Model theoretically (Carrera et al., 2004; Jin et al., 2013; Nuhoglu and Yalcin, 2005; Sheintuch et al., 1995; Zheng et al., 2017). |

**Table S3** Specific ammonium or nitrite oxidation activity at 37℃ under their optimal media and the unified medium. Data are shown as mean ± SD (n = 5 or 4 for the unified medium or specific media, respectively).

| Pure culture | Specific oxidation activity at 37℃ (h^-1^) | |
| --- | --- | --- |
|  | Unified medium | Specific medium |
| *N. inopinata* | 0.022 ± 0.004 | 0.021 ± 0.001 |
| *N. gargensis* | 0.011 ± 0.001 | 0.010 ± 0.002 |
| *N. communis* | 0.023 ± 0.003 | 0.024 ± 0.002 |
| *N. moscoviensis* | 0.019 ± 0.004 | 0.022 ± 0.001 |

**Table S4** Analysis of similarities (ANOSIM) among N1, N2C, N2A, N2B and N4 communities at the final time point under different ammonium concentrations.

| Treatment | N1 | N2C | N2A | N2B | N4 |
| --- | --- | --- | --- | --- | --- |
| N1 |  | 0.165 | 0.001 | 0.001 | 0.001 |
| N2C |  |  | 0.001 | 0.001 | 0.001 |
| N2A |  |  |  | 0.001 | 0.001 |
| N2B |  |  |  |  | 0.001 |
| N4 |  |  |  |  |  |

**Table S5** Multiple-response permutation procedure (MRPP) analysis of N4 and other four synthetic communities at the final time point under different ammonium concentrations.

| Ammonium conc. (mM) | | MRPP results | | | |
| --- | --- | --- | --- | --- | --- |
|  |  | N1 | N2C | N2A | N2B |
| N4 | 0.2 | 0.033 | 0.026 | 0.029 | 0.026 |
|  | 1 | 0.019 | 0.028 | 0.034 | 0.036 |
|  | 2 | 0.026 | 0.027 | 0.03 | **0.064** |
|  | 10 | 0.03 | 0.025 | 0.026 | 0.03 |
|  | 20 | 0.034 | 0.019 | 0.029 | **0.787** |

**Table S6** The maximum total cell number in five ammonium concentrations

| Ammonium conc. | Max total cell number (copies/mL) |
| --- | --- |
| 0.2 mM | 1.22×10^5^±3.56×10^4^ |
| 1 mM | 3.77×10^6^±1.09×10^6^ |
| 2 mM | 1.89×10^7^±1.50×10^6^ |
| 10 mM | 1.53×10^8^±1.42×10^7^ |
| 20 mM | 3.79×10^7^±5.23×10^6^ |


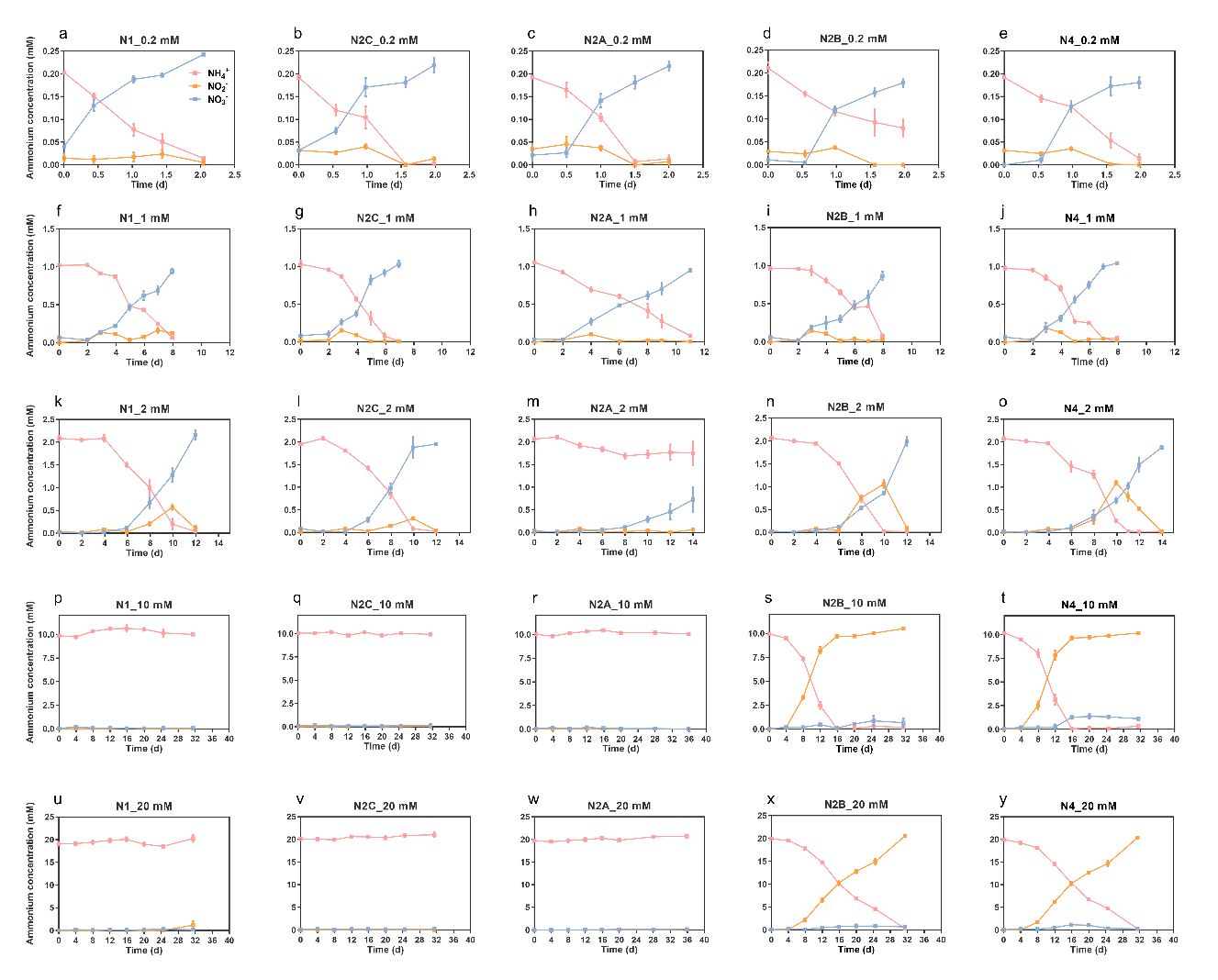
**Figure S1** The ammonium, nitrite and nitrate concentrations in N1, N2C, N2A, N2B and N4 communities under five (0.2 to 20 mM) ammonium concentrations. Data are presented as mean ± SE (n = 4).


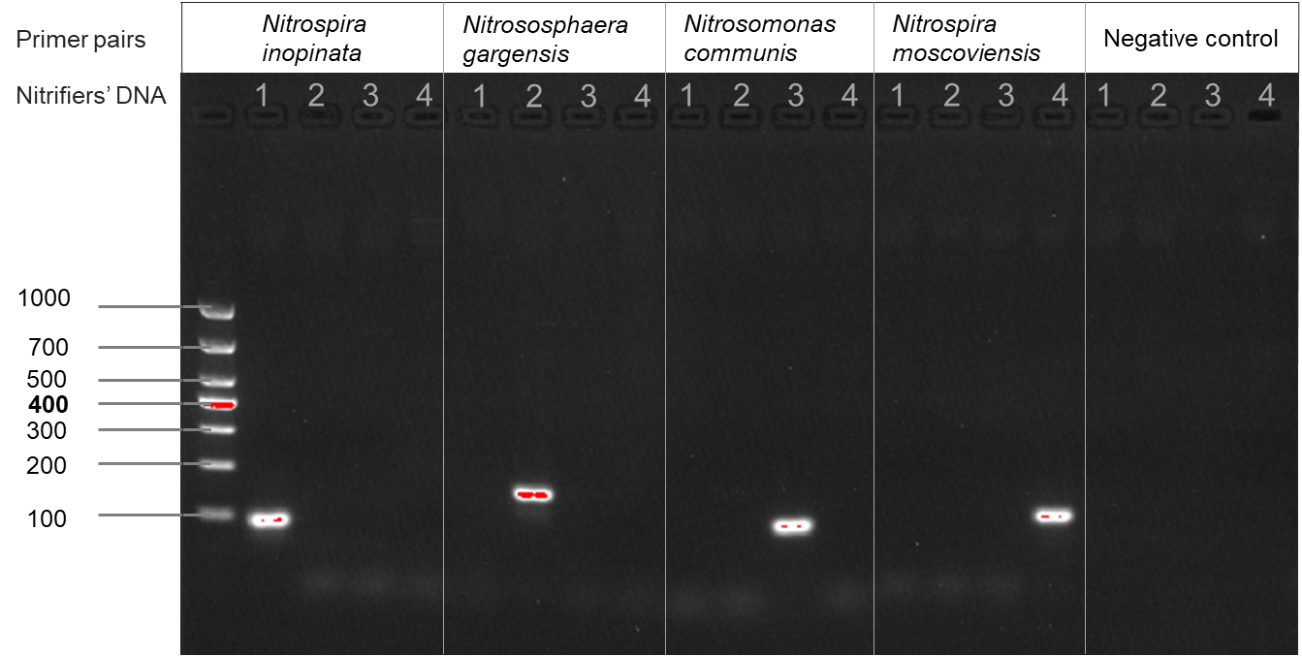


**Figure S2** The primer specificity for quantitative PCR (qPCR) analysis of four nitrifiers. The numbers 1-4 mean DNA extracted from *N. inopinata*, *N. gargensis*, *N. communis* and *N. moscoviensis*, respectively.


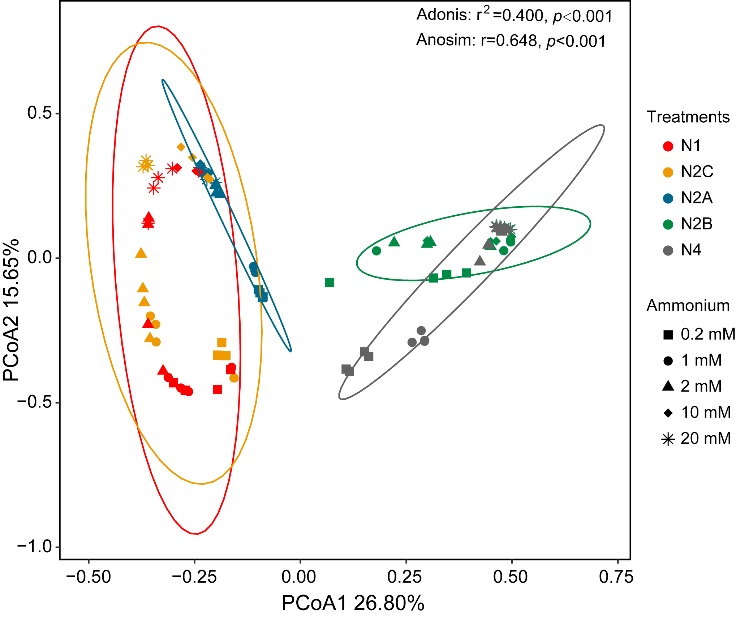


**Figure S****3** Principal co-ordinates analysis (PCoA) of synthetic nitrifying communities (N1, N2A, N2B, N2C and N4) under five ammonium concentrations at the last time point. The samples in an ellipse showed a 95% confidence within this group. The values of PCoA1 and PCoA2 labels were percentages of variations explained. Colors and shapes represent different treatments and different ammonium concentrations, respectively. The significance of dissimilarities was examined by ANOSIM or MRPP (Table S3, Table S4).


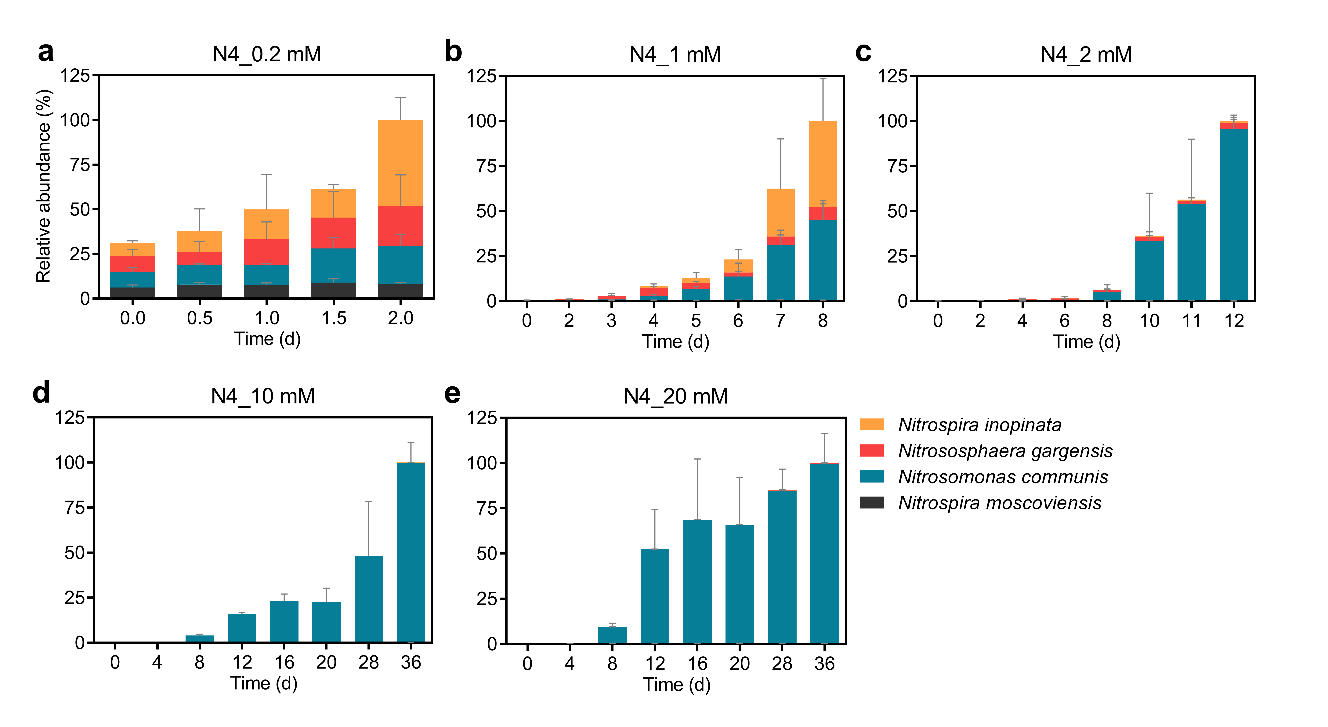


**Figure S4** Relative abundances of four nitrifiers in the N4 community analyzed by qPCR. a. 0.2 mM; b. 1 mM; c. 2 mM; d. 10 mM; e. 20 mM. In every ammonium concentration, the cell (copy) number of the N4 communities at the final time point had the highest maximum cell number and was used as 100%, the relative abundance of nitrifies at rest time point were calculated by dividing the highest maximum cell number. Data are presented as mean ± SE (n = 4).

**
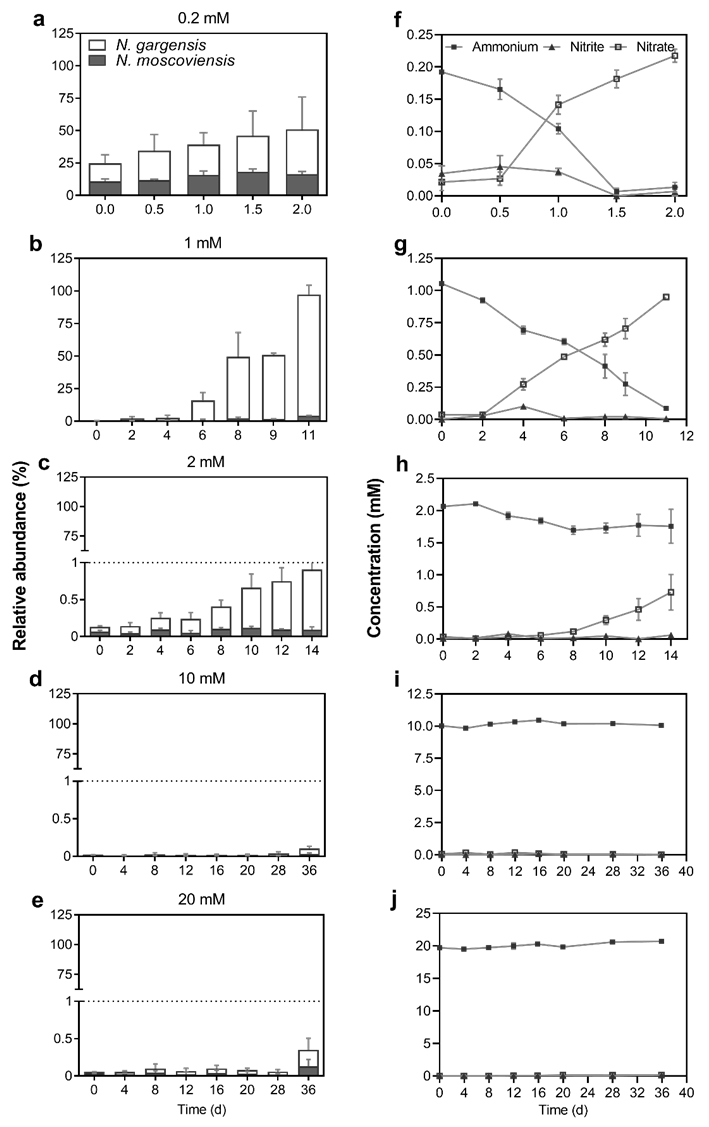
**

**Figure S5** The abundance (a-e) and ammonium oxidation (f-j) of N2A (*N. gargensis* and *N. moscoviensis*) under five (0.2 to 20 mM) ammonium concentrations. Data are presented as mean ± SE (n = 4).


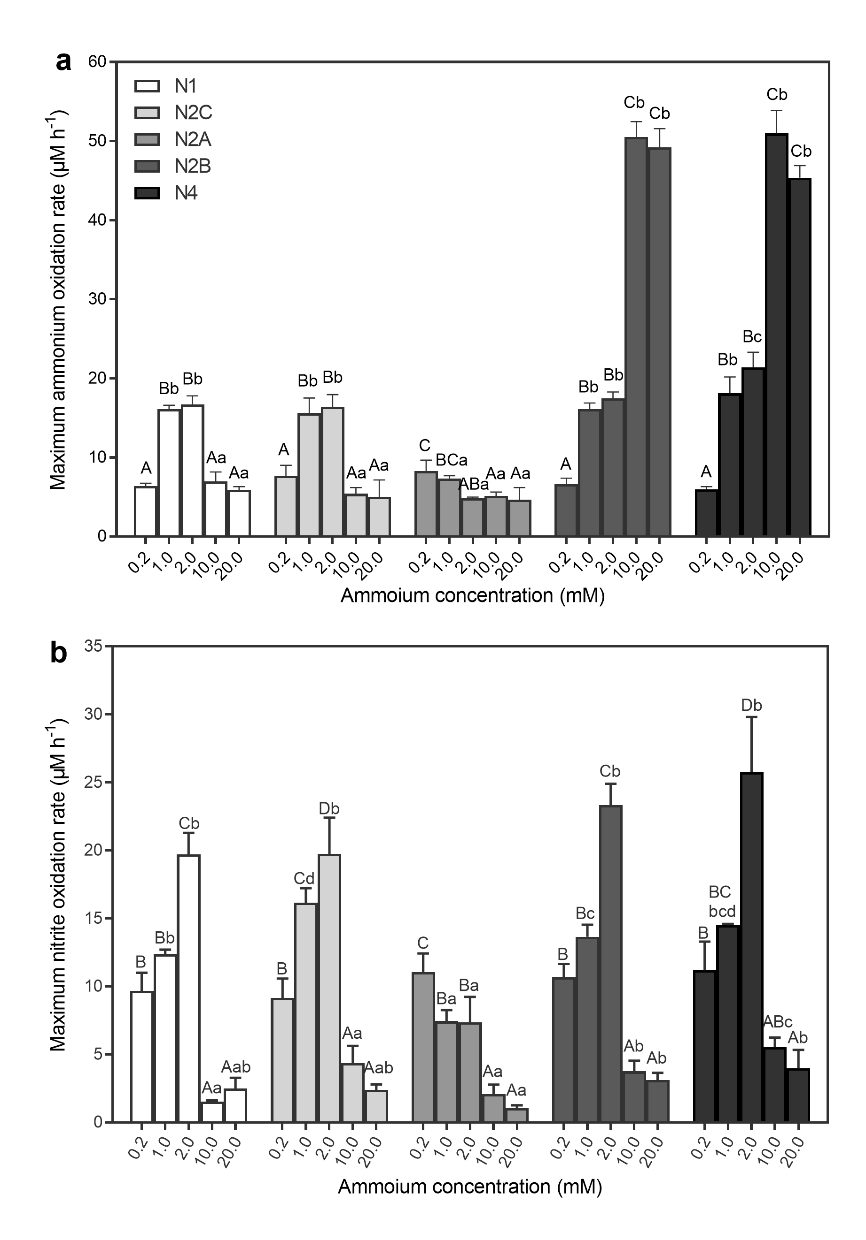


**Figure S6** Maximum ammonium and nitrite oxidation rates of five nitrifying communities at the ammonium concentrations of 0.2, 1, 2, 10 and 20 mM. Data points are shown as mean ± SE (n = 4). Different capital letters mean a statistical significance (*P*<0.05) among the five ammonium concentrations for the same community, and different small letters mean a statistical significance (*P*<0.05) among different synthetic nitrifying communities under the same ammonium concentration.

**References**

Anthonisen, A.C., Loehr, R.C., Prakasam, T.B. and Srinath, E.G. 1976. Inhibition of nitrification by ammonia and nitrous acid. Water Pollution Control Federation 48(5), 835-852.

Carrera, J., Jubany, I., Carvallo, L., Chamy, R. and Lafuente, J. 2004. Kinetic models for nitrification inhibition by ammonium and nitrite in a suspended and an immobilised biomass systems. Process Biochemistry 39(9), 1159-1165.

Jin, R.C., Xing, B.S., Yu, J.J., Qin, T.Y. and Chen, S.X. 2013. The importance of the substrate ratio in the operation of the Anammox process in upflow biofilter. Ecological Engineering 53, 130-137.

Kits, K.D., Sedlacek, C.J., Lebedeva, E.V., Han, P., Bulaev, A., Pjevac, P., Daebeler, A., Romano, S., Albertsen, M., Stein, L.Y., Daims, H. and Wagner, M. 2017. Kinetic analysis of a complete nitrifier reveals an oligotrophic lifestyle. Nature 549(7671), 269-272.

Nuhoglu, A. and Yalcin, B. 2005. Modelling of phenol removal in a batch reactor. Process Biochemistry 40(3-4), 1233-1239.

Prosser, J.I. and Nicol, G.W. 2012. Archaeal and bacterial ammonia-oxidisers in soil: the quest for niche specialisation and differentiation. Trends in Microbiology 20(11), 523-531.

Sheintuch, M., Tartakovsky, B., Narkis, N. and Rebhun, M. 1995. Substrate inhibition and multiple states in a continuous nitrification process. Water Research 29(3), 953-963.

Zheng, Z., Li, J., Ma, J., Du, J., Wang, F., Bian, W., Zhang, Y. and Zhao, B. 2017. Inhibition factors and Kinetic model for ammonium inhibition on the anammox process of the SNAD biofilm. Journal of Environmental Sciences 53, 60-67.
